# An *in vitro* Comparative Evaluation of Efficacy of Disinfecting Ability of Garlic Oil, Neem Oil, Clove Oil, and Tulsi Oil with autoclaving on Endodontic K Files tested against *Enterococcus faecalis*

**DOI:** 10.5005/jp-journals-10005-1451

**Published:** 2017-02-27

**Authors:** Shivayogi Hugar, Punit M Patel, Jyoti Nagmoti, Chaitanya Uppin, Laresh Mistry, Neha Dhariwal

**Affiliations:** 1 Professor, Department of Pedodontics and Preventive Dentistry KLE VK Institute of Dental Sciences, KLE University, Belagavi Karnataka, India; 2 Postgraduate Student, Department of Pedodontics and Preventive Dentistry KLE VK Institute of Dental Sciences, KLE University, Belagavi Karnataka, India; 3 Professor, Department of Microbiology, Jawaharlal Nehru Medical College KLE. University, Belagavi, Karnataka, India; 4 Lecturer, Department of Pedodontics and Preventive Dentistry KLE VK Institute of Dental Sciences, KLE University, Belagavi Karnataka, India; 5 Lecturer, Department of Pedodontics and Preventive Dentistry, Mahatma Gandhi Mission’s Dental College and Hospital, Navi Mumbai Maharashtra, India; 6 Postgraduate Student, Department of Pedodontics and Preventive Dentistry KLE VK Institute of Dental Sciences, KLE University, Belagavi Karnataka, India

**Keywords:** Autoclave, Clove oil, Disinfection, *Enterococcus faecalis*, Garlic oil, Neem oil, Tulsi oil.

## Abstract

**Aim:**

To comparatively evaluate the efficacy of disinfecting ability of garlic oil, neem oil, clove oil, and tulsi oil with autoclaving on endodontic K files tested against *Enterococcus faecalis.*

**Materials and methods:**

Fifty endodontic K files were exposed to the test micro-organism and checked for its disinfecting ability using three different methods.

**Result:**

Garlic oil, clove oil, tulsi oil and autoclave showed considerable effectiveness against *E. faecalis* except neem oil.

**Conclusion:**

Garlic oil, clove oil and tulsi oil are an effective disinfectant and can be used as an alternative to autoclaving against the test micro-organism.

**Clinical Significance:**

Herbs and herbal extracts are a natural and harmless way of controlling infection. These products are readily available and comparable to gold standard, thus can have its applications in rural India.

**How to cite this article:**

Hugar S, Patel PM, Nagmoti J, Uppin C, Mistry L, Dhariwal N. An *in vitro* Comparative Evaluation of Efficacy of Disinfecting Ability of Garlic Oil, Neem Oil, Clove Oil, and Tulsi Oil with autoclaving on Endodontic K Files tested against *Enterococcus faecalis.* Int J Clin Pediatr Dent 2017;10(3):283-288.

## INTRODUCTION

Microorganisms induce a variety of infections and diseases in the human body and are largely ubiquitous in the nature of the contamination, directly or indirectly leading to transmission of infectious agents.^1^ The human oral cavity contains a wide variety of microorganisms— both commensals and pathogenic organisms—and these pathogenic microorganism are the ones causing infec-tions.^2^ Due to a large number of bacteria, it becomes impossible to work in the sterile environment. Infection control procedures are essential to modern dentistry and have an impact on clinical outcomes.

Endodontic procedures are one of the most common treatments carried out in the dental operatory. Endodontic instruments are one of the "critical items" as they contact the vital tissues of the body. Endodontic files are one of them and, thus, should be sterile before use and reuse.^3^ There is a high chance of transmitting pathogenic microbes via end-odontic instruments in the absence of adequate infection control. It has been found that these instruments can even be supplied by the manufacturer with a high microbial content obviating the need to sterilize them even before their first use.^4^ Since the advent of the variant Creutzfeldt-Jakob disease infectivity, disinfection and infection control play a major role, as it is an incurable and fatal disease.^5^

Although there are many procedures to sterilize or disinfect files, they do have some shortcomings. Autoclav-ing is considered to be the gold standard for sterilization, but it has a disadvantage in that it tempers the mechanical properties of files by reducing their cutting efficiency.^6^

Of all the endodontic pathogens, *E. faecalis* is commonly found in the primary and persistent root canal infections. It is a Gram-positive, facultative anaerobic bacteria, which is resistant to various disinfection procedures.^7^

Phytodentistry has emerged with dentistry due to its properties, such as it does not tamper with physical properties and microorganisms are unable to develop resistance against the agents used.^8^ Many studies showed the efficacy of garlic oil, neem oil, clove oil, and tulsi oil against various microorganisms.^9-14^ Thus, the aim of our study was to compare the disinfecting efficacy of garlic oil, neem oil, clove oil, and tulsi oil with autoclaving against *E. faecalis.*

**Fig. 1:**
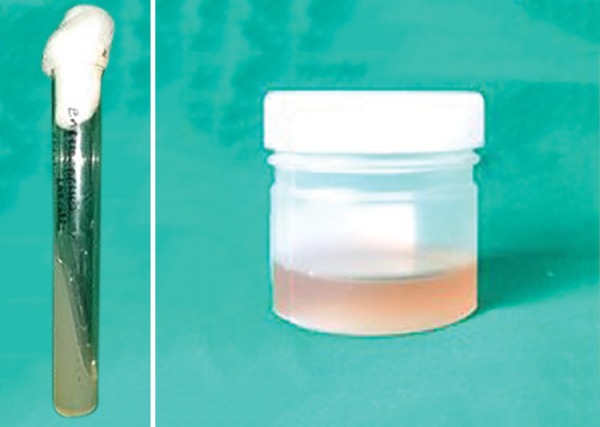
*Enterococcus faecalis* broth

**Fig. 2:**
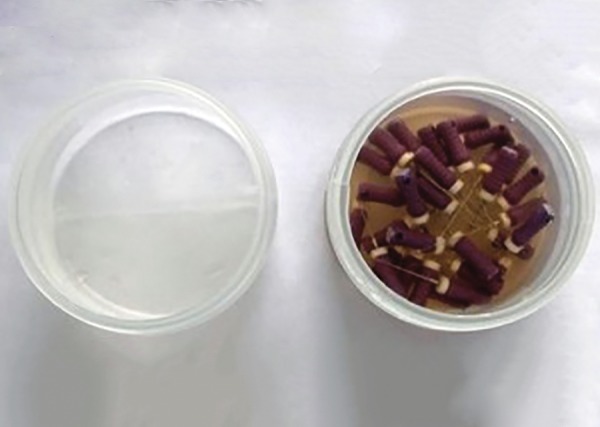
Endodontic files infected by placing in *E. faecalis* broth for 30 minutes

**Fig. 3:**
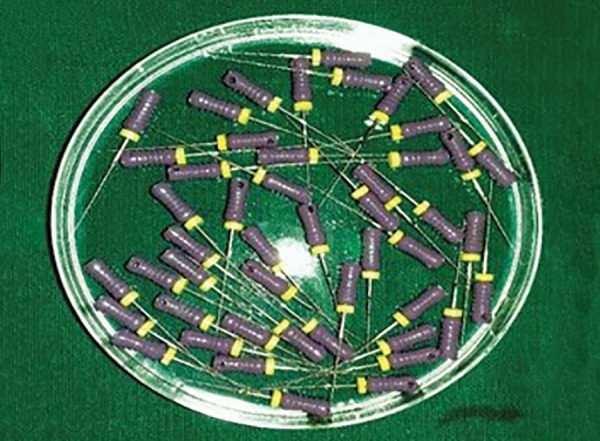
Infected endodontic files placed in petri dish

## MATERIALS AND METHODS

The study was performed on 50 K files, 25 mm long and of size 10. The significance behind using # 10 K file was that they were the first endodontic files inserted into the canal and encounter the maximum infection. All files were presterilized using standard autoclaving protocol at 121°C at 15 psi for 30 minutes. *E. faecalis* standard strain ATCC (American Type Cell Culture) 21292 was used for disinfecting the files.

The *E. faecalis* broth was prepared according to the standard microbiological protocols and incubated overnight for a period of 24 hours at 37°C (Fig. 1).

After the broth was obtained, presterilized files were infected by inserting them into a sterile container containing the broth for a period of 30 minutes (Fig. 2).

Following this, the files were transferred to a sterile petri dish and incubated for 30 minutes at 37°C for drying and ensuring fixation of the microbes on the files (Fig. 3).

The files were equally divided and inserted into the test chemicals:

- *Group I:* 10 K Files treated with 0.5% w/v garlic oil extract. (Dr. Jain’s Forest Herbals Private Limited, Andheri, Mumbai, India)
- *Group II:* 10 K Files treated with neem leaf oil. (Dr. Jain’s Forest Herbals Private Limited, Andheri, Mumbai, India)
- *Group III:* 10 K files treated with clove leaf oil. (Dr. Jain’s Forest Herbals Private Limited, Andheri, Mumbai, India)
- *Group IV:* 10 K files treated with tulsi leaf oil. (Dr Jain’s Forest Herbals Private Limited, Andheri, Mumbai, India)
- *Group V:* 10 K files treated with autoclaving at 121°C at 15 lbs for 30 minutes.

**Figs 4A to E:**
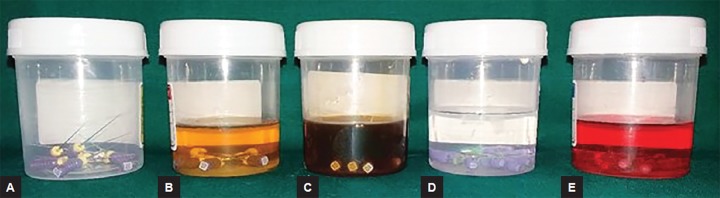
Infected endodontic files placed in: (A) Autoclave; (B) 0.5% w/w garlic oil; (C) neem leaf oil; (D) tulsi leaf oil; and (E) pure clove leaf oil

The endodontic files in groups I, II, III, and IV were placed into the test chemical for 5 minutes and group V was autoclaved according to the standard protocol (Fig. 4).

The disinfecting abilities of the test chemicals were checked using the three methods.

1. Turbidity method
2. Blood agar plate streaking method
3. Microscopic examination

**Figs 5A to F:**
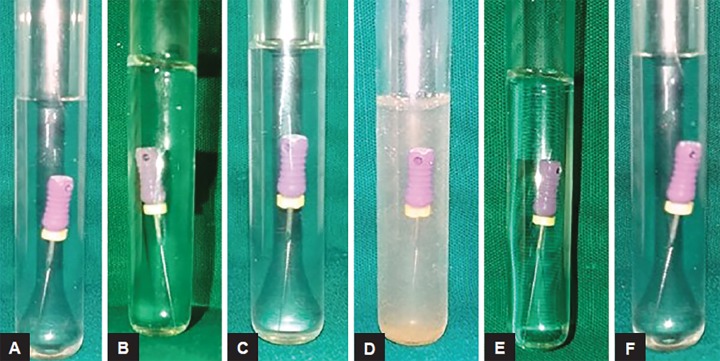
Disinfection of K files checked by turbidity method: (A) Control; (B) autoclave; (C) 0.5% w/w garlic oil; (D) neem leaf oil; (E) tulsi leaf oil; and (F) pure clove leaf oil

In the turbidity method, all the infected files were placed into 50 test tubes containing peptone water and incubated overnight to check for turbidity, which was suggestive of the growth of bacteria (Fig. 5).

**Figs 6A to E:**
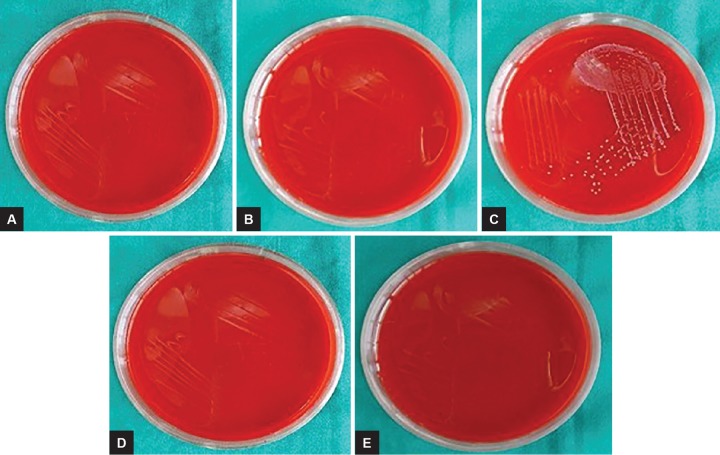
Disinfection of K files checked by blood agar plate streaking method: (A) Autoclave; (B) 0.5% w/w garlic oil; (C) neem leaf oil; (D) tulsi leaf oil; and (E) pure clove leaf oil

In blood agar streaking method, using the sterile loupes, the inoculum from the test tube was streaked onto the blood agar plate and checked for colony growth (Fig. 6).

**Figs 7A to F:**
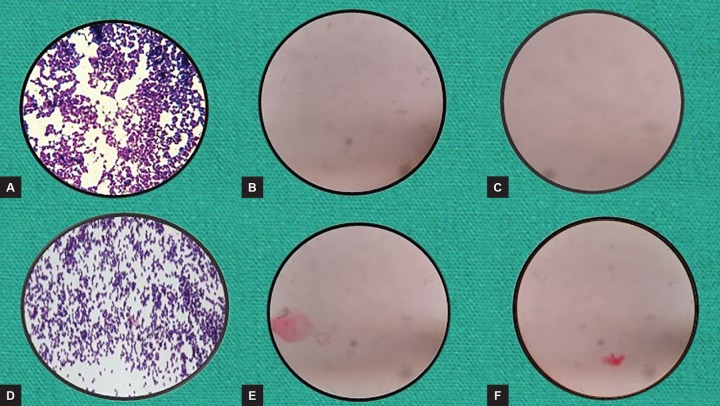
Disinfection of K files checked by microscopic examination method: (A) Control; (B) autoclave; (C) 0.5% w/w garlic oil; (D) neem leaf oil; (E) tulsi leaf oil; and (F) pure clove leaf oil

In case of microscopic examination using Gram’s stain, the standard strain was checked with respect to all disinfected files by the different methods (Fig. 7).

### Statistics

All the data were tabulated and tested statistically using one-way analysis of variance test and Chi-squared test using Statistical Package for the Social Sciences 18 software.

## RESULTS

Statistically significant result was seen between the neem oil group and other test material groups. All the endodontic files used in the study except the ones for the neem oil group showed no growth of *E. faecalis,* while the files disinfected with the neem oil group showed turbidity in peptone water, peculiar growth of *E. faecalis* on blood agar, and Gram-positive cocci colonies under microscopic examination.

## DISCUSSION

An important cause of spread of infection from one person to another is through the use of contaminated instruments. The trend in health care settings is moving toward single-use instruments. It has been suggested that endodontic files should be for single use only, but because of cost implications, this has not yet been implemented.^5^ Due to the risk of cross-contamination, resulting in the spread of dreadful disease like human immunodeficiency virus - acquired immunodeficiency syndrome, Creutzfeldt-Jacob’s disease, etc., their cleaning and sterilization are of paramount importance.

The *E. faecalis* is an important microorganism in the view of its potential to cause significant resistance to pulpal and periapical infection.^8^ It is an obligate anaerobe, which thrives well under reduced oxygen tension and is significantly resistant to commonly used intracanal medicaments and irrigating regimens as outlined by various studies.^7,9^ Thus, *E. faecalis* was used in the present study to determine the disinfecting potential of the different test chemicals.

Garlic oil has proven its antimicrobial properties by various studies.^10-13^Groppo et al^10^ showed that garlic oil was effective in reducing the oral microorganisms. Tsao and Yin^11^ found that *Pseudomonas* and *Klebsiella* which were resistant to various drugs including Ceftazidime, Gentamycin, Imipenem, and Meropenem were sensitive to garlic oil. Bakri and Douglos^12^ found that garlic oil was effective against most of the microorganisms, with *Streptococcus mutans* and *Porphyromonas gingivalis* being the most sensitive. Ruddock et al^13^ concluded that garlic oil was effective against *E. faecalis* similar to our study along with *Neisseria* and *Staphylococci* also being susceptible.

In our present study, garlic oil was found to be effective against *E. faecalis* similar to autoclave. The reason being garlic contains an active component—allicin—that is produced by the action of allinase enzyme on allin. This allicin reacts with thiol groups of enzymes in susceptible bacteria to form S-allylmercaptocysteine, thus causing their inhibition. This disrupts the metabolic activity of the bacteria and causes cell lysis.^14^

Clove oil has a long history of use as a natural antimicrobial agent. There is enough literature to state its efficacy against the oral and systemic pathogens.^15-17^ Nascimento et al,^15^ in their study, proved the efficacy of clove oil against *Staphylococcus aureus, Klebsiella pneumoniae, Pseudomonas aeruginosa,* and *Candida albicans;* when they tested other herbs, such as basil, guava, rosemary, sage, pomegranate, yarrow, jambolan, thyme, and lemon balm, clove oil showed to be the most effective. Clove oil was found to be more effective when compared with clove extract and scorbic acid against the foodborne pathogens.^16^ Hugar et al^17^ showed that clove oil was effective against *E. faecalis,* which was in accordance to our study.

Clove oil has (70-90%) eugenol.^18^ This causes sensiti-zation of the phospholipid bilayer of the microbial cyto-plasmic membrane causing increased permeability and unavailability of vital intracellular constituents resulting in disruption of the cell membrane leading to leakage of cellular constituents and finally bacterial cell death.^17^

Tulsi oil has been widely investigated due to its easy availability and antimicrobial properties. Fine et al^19^ proved that tulsi oil reduced almost 75.4% *S. mutans* present in the plaque sample. According to Agrawal and Nagesh,^20^ tulsi extract demonstrated an antimicrobial activity against *Streptococcus mutans* and showed maximum antimicrobial potential at the 4% concentration. Tulsi oil was moderately effective against the planktonic and biofilm forms of *E. faecalis.^21^* In our study, it was seen that tulsi oil was effective against *E. faecalis;* similar results were found by Subbiya et al^22^ where tulsi oil significantly reduced the *E. faecalis* count present in the dentinal biofilm.

The active constituents of tulsi are tannins (4.6%) and essential oil (up to 2%). The essential oil consists principally of eugenol (up to 62%) and methyl-eugenol (up to 86%). The antimicrobial action of tannins is due to their ability to form complexes with enzymes or substrates required by microorganisms for their functioning or may be related to its action on the cell membrane of the microorganisms.^22^

Neem is commonly seen as a medicinal tree and versatile plant having a wide spectrum of biological activities. Various studies have been put forward on its antimicrobial properties. A study by Elavarasu et al^23^ stated that neem oil was effective by reducing the growth of the plaque-causing microorganisms. Bohora et al^24^ found that neem extract showed significant antimicrobial effect against *E. faecalis* and *C. albicans.* But a study by Kumar and Sidhu^25^ stated that neem extract was ineffective and did not inhibit the growth of *E. faecalis;* similar result was obtained in the present study.

Neem contains terpenoids like nimbin, flavonoids, tannins, and alkaloids. These phytochemicals act on bacterial cell wall by binding to adhesins forming complex causing inactivation of proteins and cell function.^26^

Autoclave is considered to be a gold standard and is recommended as an ideal method of sterilization, as it results in complete eradication of spores and microorganisms. It works on the principle of coagulation and dena-turation of essential proteins structures resulting in cell lysis.^27^ But, it has a drawback of tempering the mechanical properties of the instruments. Study by Neal et al^6^ proved that autoclave caused reduction in the cutting ability of the endodontic files.

Thus, our present study concludes that garlic oil, clove oil, and tulsi oil are as effective as autoclave for disinfection and can be considered as an alternative to it, but neem oil was found to be ineffective.

### Advantages

It requires less chairside time, is economical, easily available, with minimal possibility of developing resistance, does not temper the physical properties of files, and does not require electrical supply and can be carried out in the rural areas.

### Limitations of Our Study

An *ex vivo* study needs to be carried out to check for their efficacies on the *in vivo* strains. A large sample size with a wider spectrum of bacteria needs to be tested to validate its use.

## CONCLUSION

Herbs and herbal extracts are a natural and harmless way of controlling infection. These products are readily available and are comparable with the gold standard and can have their applications in rural parts of India. Clove oil, garlic oil, and tulsi oil have excellent antibacterial properties and can be used for endodontic infection control.
